# Transiently Expressed Mistletoe Lectin II in *Nicotiana benthamiana* Demonstrates Anticancer Activity In Vitro

**DOI:** 10.3390/molecules25112562

**Published:** 2020-05-31

**Authors:** Milena Mazalovska, J. Calvin Kouokam

**Affiliations:** 1 Department of Pharmacology and Toxicology, University of Louisville School of Medicine, Louisville, KY 40202, USA; milena.mazalovska@louisville.edu; 2 Center for Predictive Medicine, University of Louisville School of Medicine, Louisville, KY 40202, USA; 3 James Graham Brown Cancer Center, University of Louisville School of Medicine, Louisville, KY 40202, USA

**Keywords:** mistletoe lectin-II, transient expression, *Nicotiana benthamiana*, anticancer, apoptosis

## Abstract

Mistletoe (*Viscum album*) extracts have been used as alternative and complementary therapeutic preparations in multiple cancers for decades. Mistletoe lectins (ML-I, ML-II, and ML-III) are considered to be the main anticancer components of such preparations. In the present study, ML-II was transiently expressed in *Nicotiana benthamiana* using the pEAQ-HT expression system. Expression levels of up to 60 mg/kg of the infiltrated plant tissue were obtained, and a three-fold increase was achieved by adding the endoplasmic reticulum (ER) retention signal KDEL to the native ML-II sequence. The native protein containing His-tag and KDEL was purified by immobilized metal affinity chromatography (IMAC) and gel filtration. We found that the recombinant ML-II lectin was glycosylated and retained its carbohydrate-binding activity. In addition, we demonstrated that plant produced ML-II displayed anticancer activity in vitro, inhibiting non-small cell lung cancer H460 and A549 cells with EC50 values of 4 and 3.5 µg/mL, respectively. Annexin V-448A and PI double staining revealed that cell cytotoxicity occurred via apoptosis induction. These results indicate that ML-II transiently expressed in *N. benthamiana* plants is a promising candidate as an anticancer agent, although further optimization of production and purification methods is required to enable further in vitro testing, as well as in vivo assays.

## 1. Introduction

Cancer remains a leading cause of death worldwide with an estimated 9.6 million deaths in 2018 [1]. According to the American Cancer Society, more than 1.8 million new cancer diagnoses are expected with 606, 520 cancer related deaths in 2020 [2]. In the USA, cancer is the second most common cause of death only after heart disease [3]. The main malignancy is lung cancer, followed by breast, prostate, and colorectal cancers based on CDC cancer statistics [4].

Cancer treatment typically includes surgery, chemotherapy and/or radiotherapy, and more recently immunotherapy [5,6,7]. Despite major improvements over the years in cancer therapy and early diagnosis, cancer patients still opt for complementary treatment to reduce the pain associated with conventional therapies and to increase their quality of life [8,9]. It has been reported that more than 50% of cancer patients use complementary treatment [10].

In the last decade, standardized mistletoe (*Viscum album*) extracts have been commonly applied as complementary and alternative therapeutics in cancer, especially in Europe [11,12,13]. These preparations have been tested in clinical trials for anticancer properties, alone or in combination with existing therapies. However, their mode of action and therapeutically beneficial effects remain largely undefined. Despite being used and advocated as an effective treatment option for cancer patients, few studies have attempted to elucidate the efficacy or mode of action of mistletoe extracts, or their separate active components. Although these preparations encompass many metabolites with cytotoxic and immunomodulatory activities, mistletoe lectins (ML-I, ML-II, and ML-III) are considered to be the main anticancer components of the extract [14].

Lectins are defined as widely distributed proteins that specifically bind carbohydrate moieties without changing their structures. Their carbohydrate-binding features and sugar specificities makes them useful biotechnological tools applied as vaccine adjuvants, microbicides, or lectin arrays for glycosylation profiling [15,16]. However, lectins also have the ability to induce programmed cell death in cancer cells, making them great research objects as antitumor agents [17,18]. For example, plant lectins from the GNA-related agglutinin family, such as *Polygonatum cyrtonema* lectin (PCL) [19], *Polygonatum odoratum* (POL) [20], *Remusatia vivipara* (RVL) [21], legume family lectins such as Concanavalin A (Con A) [22], *Dioclea violacea* lectin (DVL) [23], *Dioclea lasiocarpa* lectin (DLL) [24], and Ricin B family lectins such as mistletoe [25] and Korean mistletoe [26] lectins have been shown to induce apoptosis and autophagy in cancer cells.

Mistletoe lectins (ML-I to III) are toxic proteins belonging to the ribosome-inhibiting proteins (RIPs) group II and comprise two subunits, i.e., the cytotoxic subunit A and the carbohydrate-binding subunit B linked by disulfide bonds [27]. MLs differ in their sugar-binding specificities as follows: ML-I binds specifically to d-galactose, ML-III to n-acetyl-d-galactosamine, and ML-II to both sugars [28]. The three ML isoforms are a result of a post-translational processing of ML-I to ML-II and ML-III. Similar to all RIPs, MLs exert their cytotoxic effects by binding via the B chain to the cell surface and delivering the toxic subunit inside the cell, which hydrolyzes the n-glycosidic bond between adenine-4324 and guanine-4325 in 28S rRNA of the 60S subunit of ribosomes, inhibiting protein synthesis [25].

Whole mistletoe extracts are applied in clinical practice but too little is currently known about the activities of the main active components, which are compounded with the differences between the various mistletoe preparations. In addition, it has been reported that some mistletoe extracts contain lower amounts of ML-I as compared with ML-II and ML-III while others do not contain any ML-I [29]. Thus, the objective of this study was to produce mistletoe lectin II in *N. benthamiana* plants using the transient expression vector pEAQ-HT, to purify the lectin by chromatography and to determine its biochemical and in vitro anticancer properties.

## 2. Results

### 2.1. Cloning and Expression of ML-II in N. benthamiana

**Figure 1:**
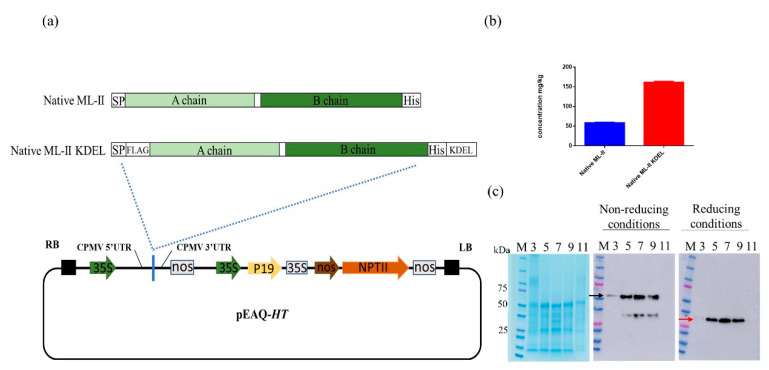
Transient expression of mistletoe lectin II (ML-II) in *Nicotiana benthamiana* via pEAQ-HT cloning. (**a**) Schematic representation of the constructs expressing native ML-II with a C-terminal His-tag and ML-II with the KDEL retention signal using the pEAQ-HT vector. The signal peptide (SP) and the His-tag (His) on the ML-II sequence are indicated in white boxes. The cauliflower mosaic virus (CaMV) 35 promoters are indicated by green arrows, whereas nopaline synthase and CaMV 35S terminators are indicated by white boxes. RB and LB represent the left and right T-DNA borders, respectively; (**b**) ELISA measuring the expression levels of the two constructs at 7 dpi; (**c**) Expression profile of the ML-II KDEL protein in *Nicotiana benthamiana*. SDS-PAGE and Western blot assessment of the total soluble protein extracted at days 3, 5, 7, 9, and 11 post infiltration, respectively. Lane 1, M protein molecular weight marker. ML-II was detected using KPL’s HisDetector. The black arrow indicates the whole protein under nonreducing conditions (50 kDa), with the red arrow showing the B chain under reducing conditions (30 kDa).

The expression construct pEAQ-ML-II was designed to produce the native ML-II protein (native signal peptide and native linker between the A and B chains) with the addition of a C-terminal His-tag to ease purification and detection (Figure 1a). This construct yielded approximately 60 mg/kg FWT and adding the ER retention signal KDEL at the C-terminus helped to improve ML-II expression by three-fold, with 160 mg/kg FWT recorded (Figure 1b). Because of elevated expression, ML-II KDEL was selected for subsequent experiments. Partial assembly of the whole protein was observed with the expression of both sequences. Several attempts were made to further increase ML-II yield and possibly guide the production process towards the expression of the whole protein versus single chains, for example, by adding different signal peptides and linkers. However, partial assembly remained, suggesting that a small portion of the protein could be cleaved in planta (data not shown). Another tag (FLAG-tag) was added at the n-terminus after the native signal sequence for purification of the whole native protein, and thus removing the single chains (Figure 1a).

To estimate the optimal harvest time, leaves from several plants were collected at days 3, 5, 7, 9, and 11 post infiltration, respectively. Analysis of the clarified extracts revealed increasing amounts of mistletoe lectin II until 7 dpi (Figure 1c). Western blot under nonreducing conditions revealed the whole protein (approximately 50 kDa) and the B chain (approximately 30 kDa), whereas a single band at 30 kDa was observed under reducing conditions. Of note, no symptoms of necrosis of the plant tissue was observed during the expression process.

### 2.2. Purification of the Native ML-II KDEL Protein

**Figure 2:**
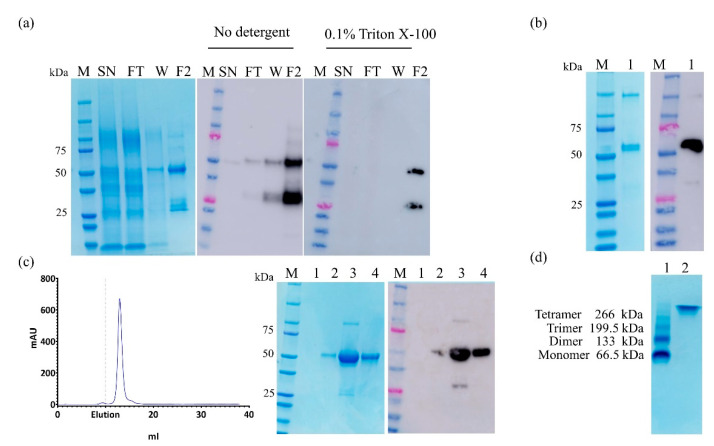
Purification of plant produced ML-II. (**a**) Purification of ML-II KDEL on immobilized metal ion affinity chromatography (IMAC). Fractions were analyzed by SDS-PAGE and Western blot. Lane 1, M protein molecular weight marker; Lane 2, clarified crude extract (SN); Lane 3, flow-through (FT) of proteins applied to the TALON column; Lanes 4, TALON resin wash fractions (W); Lanes 5, elution fractions with and without addition of detergent; (**b**) Second step purification by anti-FLAG M2 affinity resin. Lane 1, purified ML-II analyzed by SDS-PAGE and Western blot. The SDS-PAGE gel shows two bands, and the whole ML-II protein and the higher molecular band were confirmed to be mostly ML-II by LC/MS; (**c**) Purification of ML-II by gel filtration using Superdex™ 75 10/300GL column at pH 7.4 (PBS). The chromatogram of the elution profile of the ML-II protein was recorded at 280 nm. The beginning of the elution is indicated by dotted lines. Fractions 1, 2, 3, and 4 were analyzed by SDS-PAGE and Western blot, as well as native-PAGE; (**d**) Lane 1, bovine serum albumin (BSA) was used as a positive control with the respective sizes of a monomer, dimer, trimer, and tetramer. Lane 2, purified ML-II.

For large scale analysis, leaves infiltrated with the recombinant vector pEAQ-ML-II KDEL were harvested at 7 dpi as the optimal time for expression (Figure 1c). The first purification step involved metal affinity chromatography (IMAC) on the TALON resin. The protein did not completely bind to the column and could be detected in the flow through. Therefore, large amounts of the expressed protein were lost during this step. As shown in Figure 2a, adding 0.1% detergent after extraction improved ML-II binding to the TALON column. While the whole protein was observed at 50 kDa, another band corresponding to the B chain was found with HisDetector by Western blot. Since the His-tag is at the C-terminus of the protein, only the whole protein and the B chain could be detected, and both were purified simultaneously. The purity of the whole protein was calculated to be 66% by densitometric analysis of the destained gel with the Gel analyzer software. Such low purity was explained by coelution of the B chain. To separate these two proteins, the FLAG-tag was added to the n-terminus and subsequent purification using the anti-FLAG M2 affinity resin was successfully performed (Figure 2b).

To assess the identities of the 50 kDa protein and the band with higher molecular weight, these bands were excised and analyzed by LC/MS. The results confirmed that both bands were indeed ML-II, suggesting that the higher molecular weight band was an oligomer of ML-II. Due to the folding of the protein and possibly the position of the FLAG-tag at the n-terminus, this second step only retained 10% of the total ML-II protein (data not shown). We further applied gel filtration chromatography on Superdex™ 75 10/300GL as a second step of purification, and native ML-II was obtained at an estimated purity of 91.5% (Figure 2c). The expression levels obtained for the native ML-II KDEL sequence were approximately 160 mg/kg FWT, as shown above; after the gel filtration step, the protein was obtained at a yield of about 17–18 mg/kg FWT. The low yield could be increased by applying different buffers and conditions of extraction and/or purification. As shown in Figure 2d, the SDS-PAGE assessment under native conditions revealed a higher molecular weight band, confirming gel filtration data, in which the protein was not retained on the resin, and was detected in the first fractions (Figure 2c). As a reference, bovine serum albumin which is a protein with similar molecular weight that forms oligomers, was run as a positive control in native-PAGE (Figure 2d). 

### 2.3. Biochemical Characterization of N. benthamiana Produced ML-II 

**Figure 3:**
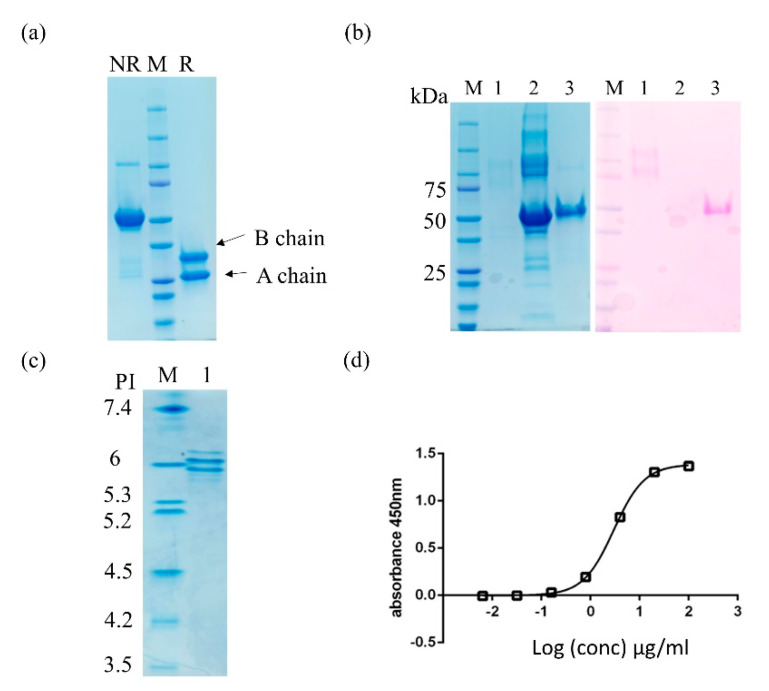
Characterization of plant produced ML-II. (**a**) SDS-PAGE of ML-II in nonreducing and reducing conditions showing the whole protein and single A (25 kDa) and B (30 kDa) chains; (**b**) SDS-PAGE with Coomassie brilliant blue and periodic acid-Schiff stained gel. Lane 1, HIV envelope glycoprotein 120 (gp120, highly glycosylated protein), Lane 2, bovine serum albumin (BSA) (a non-glycosylated protein), and Lane 3, purified ML-II; (**c**) Isoelectric focusing (IEF) of purified ML-II. Lane M, IEF marker 3–10 PI; (**d**) Carbohydrate-binding activity of ML-II by enzyme-linked lectin assay (ELLA) using asialofetuin (ASF). The EC50 of ML-II was determined to be 2.98 μg/mL.

MLs are heteromeric proteins composed of a cytotoxic subunit (A chain) and a carbohydrate-binding subunit (B chain) that are covalently linked by a disulfide bond. Purified ML-II was assessed by SDS-PAGE in nonreducing and reducing conditions showing the whole protein and separate B and A chains (Figure 3a).

In addition, native ML-II is considered to be a glycosylated protein with three potential n-glycosylation sites (positions 402, 442, and 546) all in the B chain, of which only two are likely glycosylated. Although glycosylation is not required for protein assembly or function, it is important for the stability of the lectin [30]. To confirm the glycosylation status of plant produced ML-II, bovine serum albumin as a negative control (non-glycosylated protein) and HIV envelope glycoprotein 120 (gp120, heavily glycosylated) as a positive control were evaluated alongside ML-II by periodic acid-Schiff staining (PAS) after SDS-PAGE. In this assay, only glycoproteins appear reddish purple (Figure 3b). As expected, plant produced ML-II was glycosylated. In addition, the purified protein was analyzed by isoelectic focusing (IEF), which showed the isoelectric point (PI) of the protein to be around six, which is very close to the theoretically predicted value of 5.5 (Expasy compute pI/Mw tool). In addition, plant produced ML-II appeared as a mixture of protein isoforms with different PI values, possibly due to the degree of glycosylation or other modifications occurring in planta (Figure 3c). Moreover, plant produced ML-II could bind to asialofetuin with an affinity similar to that previously reported for ML-I (Figure 3d) [31].

### 2.4. In Vitro Anticancer Activity of Plant Produced ML-II 

**Figure 4:**
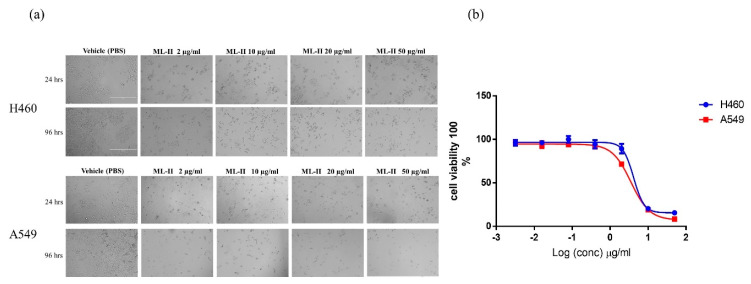
ML-II inhibits the growth of two human lung cancer cell lines in vitro. (**a**) ML-II treatment of H460 and A549 cells resulted in altered cell morphology. Cells were treated with vehicle (PBS) or ML-II (2, 10, 20, and 50 µg/mL) for 24–96 h and observed under an inverted EVOS microscope (10×). Images are representative of three independent experiments performed in triplicate (bar, 400 μm); (**b**) Cell viability measured by the 3-(4, 5-dimethylthiazol-2-yl)-5-(3-carboxymethoxy-phenyl)-2-(4-sulfophenyl)-2H-tetrazolium (MTS) cell viability assay after 96 h of incubation with ML-II or vehicle (PBS). Cells were plated in 96-well plates and treated with vehicle or ML-II, starting at a concentration of 50 µg/mL and 1/3 dilution. After 96 h, cells were incubated with the MTS reagent, and absorption was read at 490 nm. The EC50 values for H460 and A549 cells were determined to be 4 and 3.5 µg/mL, respectively. Data are expressed as percentage of the control (considered 100%), and values were presented as mean ± SD.

Treatment with ML-II induced morphological alterations in both A549 and H460 cell lines, characterized by spherical morphology of the cells and detachment from the culture surface (Figure 4a). These changes were visible as early as 24 h after treatment.

**Figure 5:**
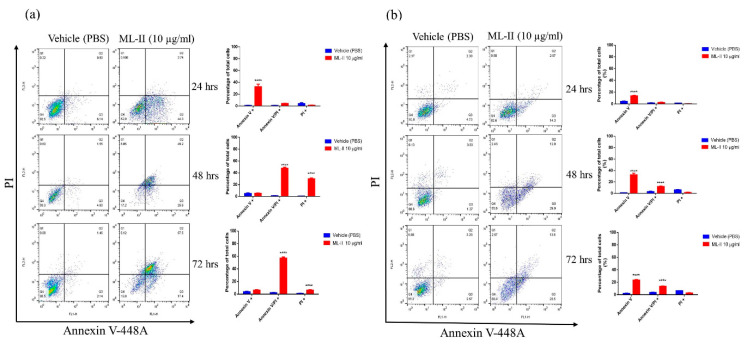
ML-II causes lung cancer cell apoptosis. (**a**) Flow cytometry data after annexin V-448Aand PI double staining of human H460 cells treated with ML-II (10 µg/mL); (**b**) Flow cytometry data after annexin V-448A and PI double staining of human A549 cells treated with ML-II (10 µg/mL). The vehicle (PBS) was used as a control, and percentages of stained cells were assessed at 24, 48, and 72 h after treatment. The right lower quadrant represents annexin V-448A positive/propidium iodide (PI) negative staining (Q1-LR: AV+/PI−) indicating early apoptosis; the right upper quadrant represents both high annexin V-448A and PI staining (Q1-UR: AV+/PI+) indicating late apoptosis; the left upper quadrant represents low annexin V-448A, and high PI staining (Q1-UL: AV−/PI+) indicating necrosis; and the left lower quadrant (Q1-LL: AV−/PI−) indicates viable cells.

Higher concentrations and longer incubation periods resulted in increased debris from dying cells. The results of the 3-(4, 5-dimethylthiazol-2-yl)-5-(3-carboxymethoxy-phenyl)-2-(4-sulfophenyl)-2H-tetrazolium (MTS) viability assay at 96 h after treatment indicated that ML-II decreased viability in both cell lines in a similar dose-dependent manner. The EC50 values for H460 and A549 cell lines were 4 µg/mL and 3.5 µg/mL, respectively (Figure 4b). The apoptotic effect of ML-II was next assessed in both cancer cell lines by annexin V-448A and PI double staining. Cells were treated with 10 µg/mL ML-II, and cell death was quantified at different time points (24–72 h). The results demonstrated an increased early apoptotic death after 24 h of treatment that progressed to late apoptosis at 48 and 72 h in both cell lines as compared with the vehicle (PBS) group (Figure 5a,b). 

**Figure 6:**
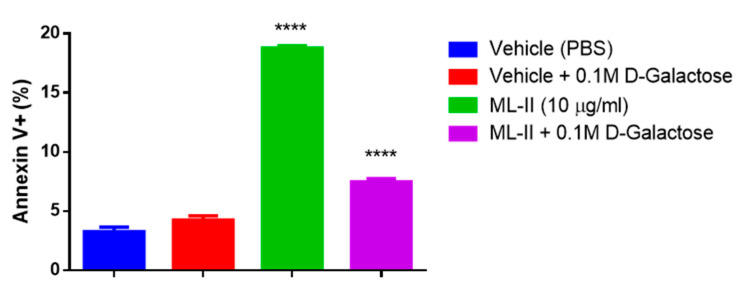
ML-II-induced apoptosis is reduced by incubation with d-galactose. Flow cytometry data after annexin V-448A and PI double-staining of H460 cells administered vehicle (PBS), PBS + 0.1 M galactose, ML-II (10 µg/mL), and ML-II (10 µg/mL) + 0.1 M d-galactose. Percentages of annexin V-448A positive cells were assessed after 24 h. Groups were analyzed by one-way ANOVA using Bonferroni’s multiple comparison test (*p* < 0.0001). Asterisks (****) indicate statistically significant differences (*p* < 0.0001) for the vehicle group vs. ML-II group and ML-II group vs. ML-II + 0.1 M galactose group. ML-II + 0.1 M galactose vs. ML-II showed >50% apoptosis reduction.

To confirm that the observed apoptotic effect was due to the d-galactose-specific lectin, H460 cells were treated with the vehicle (PBS), ML-II (10 µg/mL), and ML-II pre-incubated (1 h at 37 °C) with 0.1 M d-galactose, respectively. After 1 h of incubation, the medium was replaced, and apoptotic rates were measured by flow cytometry after 24 h (Figure 6). 

Apoptosis induction by ML-II was markedly reduced after lectin incubation with d-galactose (>50%), suggesting that the galactose-specific lectin was responsible for the observed apoptotic effect.

## 3. Discussion

Currently, mistletoe extracts constitute one of the most prescribed and applied alternative and complementary treatments against cancer in German speaking countries [32,33]. The main reason for using whole extracts is the assumed synergistic therapeutic effects of the extract components themselves or of these compounds with conventional anticancer drugs [34,35]. Since the mistletoe plant grows on a variety of trees, depending on the host tree, harvesting time, and extraction procedure, the preparations vary with regard to active components and biological properties, making it difficult to assess the anticancer effects and the underlying pharmacological and molecular mechanisms. Research on the anticancer activities of single components has focused solely on mistletoe lectin I, which is also the only mistletoe lectin recombinantly produced in *E. coli* and recently in plant-cell packs and *Nicotiana benthamiana* at levels up to 7 mg/kg of purified product [36]. Previous studies have reported ML-I as the most potent of the three MLs in vitro; however, cytotoxicity varies among the three lectins depending on the cell line used. For example, ML-I, ML-II, and ML-III showed cytotoxicity against MOLT-4 cells (a human T-cell leukemia line) grown in serum-free conditions, with IC50 values of 70, 60, and 9 ng/mL, respectively; the ML-III cytotoxicity was about 10 times higher than that of ML-I [37]. Another study showed that MLs have similar cytotoxic effects on the sensitive human colon cancer HT29^mdr−^ cell line and different cytotoxic effects on a multidrug resistant variant HT29^mdr+^, with IC50 values of 3.1, 5.7, and 10 ng/mL for ML-I, ML-II, and ML-III, respectively [38]. Therefore, there is a need for further studies to provide novel insights into the cytotoxic effects of the different MLs, as well as the differential binding to various cancer cell lines. 

Here, we report, for the first-time, the transient expression of mistletoe lectin II in *N. benthamiana*. As shown above, the KDEL retention signal increased the expression levels obtained with the native ML-II sequence by three-fold to yield 160 mg/kg, and 17–18 mg/kg infiltrated tissue of pure protein was obtained. In addition, using the transient expression pEAQ-HT vector and KDEL retention signal, we achieved 2.5× higher yield as compared with that reported for ML-I expressed using the binary *Agrobacterium* pTRA vector. Partial assembly of the whole protein was observed with both pEAQ-ML-II and pEAQ-ML-II KDEL constructs, as recently reported with ML-I [36]. Similar results were also observed for mistletoe lectins purified from the native plants [39]. We demonstrated that plant produced ML-II was glycosylated and retained its carbohydrate binding activity. In addition, treatment with ML-II reduced cell viability in two human lung cancer cell lines, including A549 and H460 cell lines, with similar EC50 values, and further confirmed that the ML-II mode of action is mostly through apoptosis. Moreover, we found that ML-II formed higher molecular weight complexes after purification, but still retained its anticancer activity in vitro. It has been previously shown that ML-I isolated from mistletoe extracts at a low concentration occurred as monomer but promoted dimer formation at high levels [39]. This is not surprising since mistletoe lectins are highly hydrophobic proteins, which also explains the difficulty in achieving high expression in plants or tedious purification. Issues with aggregation and expression, or purification yield could potentially be solved by adding agents that promote protein solubility, or different buffer systems and detergents. Since ML-II is composed of two subunits connected with a disulfide bond, expression of ML-II in *E. coli* requires the expressions of A and B chains separately that need to be resolubilized before refolding steps to assemble the whole lectin. This greatly increases the cost and complexity of ML-II production in bacterial systems [36]. The ability to produce recombinant ML-II in plants without the need for solubilization and complex refolding steps provides an easier and possibly less expensive alternative to production in *E. coli* [40] or similar systems. 

In conclusion, production of recombinant ML-II in *N. benthamiana* plants via the pEAQ-HT expression vector provides an opportunity for achieving high yield and enables the assessment of its anticancer activity, as well as possible therapeutic potential in the future. Further testing of ML-II activity towards other types of cancer cell lines and primary cancer cells, as well as in vivo efficacy and safety assays in rodent models are warranted to further characterize this lectin and determine its significance as an anticancer drug.

## 4. Materials and Methods

### 4.1. Chemicals

All chemicals used in this work were purchased from WVR International (Radnor, PA, USA) unless otherwise stated. All chemicals were of analytical grade.

### 4.2. Cloning 

The coding sequence of the native mistletoe lectin II from the European mistletoe plant *V. album* (GenBank accession number AY377892.1) was synthesized as DNA fragments (Gene Universal, Newark, DE, USA). In order to increase in planta expression levels, the Kozak sequence [41] at the n-terminus and the ER retention signal KDEL at the C-terminus were added to the native ML-II sequence (Figure 1a). In addition, an n-terminal FLAG-tag was added after the signal peptide, as well as a C-terminal His-tag to improve purification and detection. Partial assembly was observed with the expression of the engineered native ML-II sequences. To address this issue and possibly increase the expression levels, we used another signal sequence (α-amylase signal peptide) and different linkers between the A and B chains (such as flexible linker and Furin cleavage site), none of which was satisfactory. Changing the original linker with a non-cleavable linker also resulted in partial assembly (data not shown). All DNA fragments were first cloned in the pGEM^®^-T vector (Promega, Madison, WI, USA), according to the manufacturer’s instructions. Next, the restriction enzymes *Age I* and *Xho I* were used to incorporate the gene fragments in the pEAQ-HT vector [25]. The two ML-II pEAQ-HT constructs were propagated in *E. coli* DH5α cultured in Luria-Bertani (LB) broth (5 g/L yeast extract, 10 g/L tryptone, and 10 g/L sodium chloride; pH 7.0) supplemented with 50 µg/mL kanamycin at 37 °C. Following transformation of competent *E. coli*, putative colonies harboring pEAQ-ML-II and pEAQ-ML-II KDEL, respectively, were screened and verified by sequencing (CGeMM DNA Facility Core, UofL). The recombinant vectors were transformed into the electrocompetent *A. tumefaciens* strain GV3103. After electroporation at 2.5 kV, the Super Optimal Broth with catabolite repression (SOC) medium was immediately added, and the cells were left to recover for 1 h at 28 °C. Then, the cells were plated on LB agar containing 50 µg/mL rifampicin and 50 µg/mL kanamycin.

### 4.3. Plant Growth Conditions and Agroinfiltration

*N. benthamiana* plants were grown in a plant growth room maintained at 25 °C and watered three times a week. Supplemental lighting was provided to maintain 16 h of daylight. In this study, 3–4-week-old plants were used for transient expression experiments. Inoculated liquid cultures of *A. tumefaciens* were grown at 28 °C in a shaking incubator for 24 h in LB medium containing rifampicin and kanamycin. *N. benthamiana* leaves were agroinfiltrated via a syringe at an optical density at 600 nm of 0.3. 

### 4.4. Protein Extraction and SDS-PAGE

Small-scale extraction was used for assessing protein expression and accumulation. Approximately 20 g of infiltrated tissue (3 plants) per time point were harvested and homogenized in a blender with 2× volume of extraction buffer (20 mM sodium phosphate buffer, pH 7.4, 150 mM NaCl, 10% glycerol, 10 mM sodium metabisulphite, and 20 mM ascorbic acid). Samples were centrifuged at 15,000× *g* for 20 min, and the supernatant was analyzed by SDS-PAGE and Western blot, as described below. 

For large-scale extraction, leaf tissue was collected at day 7 post infiltration and processed with 2× volume of leaf fresh weight (FWT) of the above extraction buffer in a Waring blender. Large cell debris were removed by squeezing the homogenate through a layer of cheesecloth and Miracloth (EMD Millipore, Billerica, MA, USA). 

### 4.5. SDS-PAGE and Western Blot

Equal amounts of protein samples (approximately 20 µg) were analyzed on 4–15% Mini-Protean TGX precast gels (Bio-Rad, Hercules, CA, USA) under reducing and nonreducing conditions and stained with Coomassie brilliant blue. The gels were incubated overnight with destaining solution (10% acetic acid, 30% methanol, and 60% dH2O). Precision Plus protein Dual Color protein prestained standard (Bio-Rad) was used as a reference for molecular weight estimation. 

After electrophoresis, the samples were transferred onto PVDF membranes (Bio-Rad Laboratories Ltd., Hertfordshire, UK) for Western blot. Membranes were blocked with 1% (*w*/*v*) BSA in PBS containing 0.05% Tween-20 (*v*/*v*) (PBST) and incubated with KPL HisDetector^TM^ Nickel-HRP (1:10,000; SeraCare Life Sciences Inc, Milford, MA, USA) at room temperature for 1 h and washed with PBST. The emitted luminescence from the Amersham ECL™ Prime Western blotting detection reagent (GE Healthcare Life Sciences, Chicago, IL, USA) was detected on an Amersham Imager 600 (GE Healthcare Life Sciences, Chicago, IL, USA). 

### 4.6. Protein Purification

The recombinant protein from leaf extracts was purified by immobilized metal-anion chromatography (IMAC) on a TALON metal affinity column (Takara Bio USA, Inc., Mountain View, CA, USA) by fast protein liquid chromatography (FPLC) using an AKTA Purifier (Amersham Biosciences, Piscataway, NJ, USA). Briefly, 0.1% Triton-X100 was added to the clarified supernatant from the large-scale extraction. After centrifugation at 15, 000× *g* for 20 min, the clarified extracts were filtered with 0.2 µm PES vacuum filters. The column was pre-equilibrated with 20 mM sodium phosphate buffer (pH 7.4) containing 150 mM NaCl and the clarified extract was applied to the column, which was washed with 10× CV of the same buffer. The His-tagged ML-II protein was eluted in the second fraction with the above buffer supplemented with imidazole (150 mM). The second step of ML-II purification was performed by anti-FLAG M2 affinity chromatography, which proved to be inefficient. Therefore, we performed gel filtration by FPLC using the AKTA Purifier. The eluted protein fraction from the metal affinity chromatography was diafiltrated with PBS to remove imidazole and concentrate the sample. Then, the solution of ML-II (0.5 mL) was applied onto a Superdex™ 75 10/300GL column (GE Healthcare, Chicago, IL, USA) pre-equilibrated with PBS. Fractionation was carried out at a flow rate of 0.5 mL/min, and the presence of protein in various fractions was monitored at 280 nm. The major peak obtained constituted the purified lectin. The ML-II KDEL protein was purified at up to 17.5 mg/kg and detected by SDS-PAGE and Western Blot. Protein concentrations were determined on a Nanodrop 1000 (Thermo Fisher Scientific, Waltham, MA, USA) and Pierce™ BCA Protein Assay Kit (Thermo Fisher Scientific, Waltham, MA, USA).

### 4.7. Liquid Chromatography-Mass Spectrometry (LC/MS) 

Purified ML-II was run on an SDS-PAGE gel in nonreducing conditions and the two prominent bands (Figure 2b) were cut and sent to the Mass Spectrometry Core Laboratory at UofL for LC/MS analysis. Briefly, extracted gel bands were destained with 60% acetonitrile/40 mM triethylammonium bicarbonate (TEA-BC) at a ratio of 1:4 (*v*/*v*), at room temperature for 5 min, followed by two 5 min rinses with ultrapure water (18 MΩ/cm). Then, gel plugs were reduced, alkylated, and digested with trypsin, and tryptic peptides and gel plug extracts were combined, as previously described [42]. Prior to the LC-MS analysis, samples were dried with a SpeedVac, resuspended in 20 µL chromatography buffer A (2% *v*/*v* acetonitrile in water and 0.1% *v*/*v* formic acid in water). 1D-LC-MS/MS was carried out on a Proxeon EASY-nLC 1000 UHPLC equipped with an Acclaim PepMap 100 75 µm × 2 cm, nanoViper (C18, 3 µm, 100 Å) trap and an Acclaim PepMap RSLC 50 µm × 15 cm, nanoViper (C18, 2 µm, 100 Å) separating column (Thermo Fisher Scientific, Waltham, MA, USA) heated at 50 °C. Tryptic peptides were separated using a 50 min linear gradient from 0% to 45% chromatography buffer B (80% *v*/*v* acetonitrile in water and 0.1% *v*/*v* formic acid in water) at 250 nL/min. The resolved peptides were introduced by nanoelectrospray ionization with the ion transfer capillary at 225 °C and the spray voltage at 1.75 kV. Mass spectrometry data were collected on an Orbitrap Elite mass spectrometer (Thermo Fisher Scientific, Waltham, MA, USA) by the Nth Order Double Play method with scan event one yielding an FTMS MS1 scan (normal mass range; 240,000 resolution; full scan type) for 300–2000 m/z fragments with charge screened and monoisotopic precursor selection enabled. Scan event two obtained HCD ITMS MS2 scans (normal mass range; rapid scan rate; centroid data type) on up to 20 peaks with a minimum signal threshold of 5000 counts from scan event one. The raw files were recalibrated offline with Xcalibur v2.2 (Thermo Fisher Scientific, Waltham, MA, USA) using the 371.1012 *m*/*z* polysiloxane peak as an internal calibrant. The acquired LC-MS data were analyzed with Proteome Discoverer v2.3.0.523 (Thermo Fisher Scientific, Waltham, MA, USA) with the UniprotKB canonical and isoform *Nicotiana benthamiana* sequences (taxonomy ID 4100) concatenated with the ML-II (MSTOE2|ML-II_MSTOE2) sequence and the 3/4/2019 version of thegpm.org cRAP database. Data searches with SequestHT considered up to two missed trypsin (KR|P) cleavage events with the dynamic modifications oxidation (M) and acetyl (protein n-term) and the static modification carbamidomethyl (C). In the consensus step, proteins were quantified from the sum of all high confidence unique and razor peptide intensities. A proteins text file was exported from the consensus workflow results of Proteome Discoverer (Thermo Fisher Scientific, Waltham, MA, USA).

### 4.8. Endotoxin Removal by Phase Separation 

To remove endotoxins from the purified protein preparations, the samples were added to 1% Triton X-114 and chilled for 5 min on ice, followed by successive incubations for 5 min at 37 °C and 1 min at 56 °C. Then, the samples were centrifuged for 10 min at 1,200 xg, and the upper aqueous phase was collected; these steps were repeated 2–4 times with 0.5% Triton X-114 (*v*/*v*). The, the supernatants were filtered through 0.2 µm filters. For measuring endotoxin levels, Endosafe cartridges were used and read on a portable Charles River Endosafe PTS system. The endotoxin levels were less than 1 EU per mg of protein.

### 4.9. Isoelectric Focusing (IEF)

Purified ML-II was analyzed on Novex^®^ IEF gels pH 3–7 (Invitrogen, Carlsbad, CA, USA) using a XCell SureLock^®^Mini-Cell, according to the manufacturer’s instructions. Briefly, 1× IEF anode and 1× cathode buffers were added to the lower and upper buffer chambers, respectively. Then, the sample was mixed with IEF sample buffer (pH 3–7) and loaded on the IEF gel. The IEF Marker 3–10 (Thermo Fisher Scientific, Waltham, MA, USA) was used as a standard for determining the PI of the protein. The running conditions were set at 100 V for 1 h, 200 V for 1 h, and 500 V for 30 min. The gel was fixed in 12% (*w*/*v*) trichloroacetic acid (TCA) and stained with Coomassie brilliant blue G-250 (CBB G-250) in accordance with the manufacturer’s manual. 

### 4.10. Periodic Acid-Schiff Staining for Glycoprotein Detection 

After SDS-PAGE, the gels were rinsed in washing solution I (1:1 (*v*/*v*) methanol and deionized water) for 30 min, and the solution was replaced with washing solution II (3% acetic acid). After the above washing steps, the gel was incubated with periodic acid with gentle agitation for 15 min. The gel was washed again with washing solution I, twice for 5 min, followed by incubation with Schiff’s reagent for 15 min. The gel was finally washed with washing solution I and rinsed in water for 10 min before imaging.

### 4.11. Determination of Carbohydrate-Binding Specificity by Enzyme-Linked Lectin Assay (ELLA)

Flat bottom Maxisorp 96-well microtiter plates (Nunc, Roskilde, Denmark) were coated with 25 µL/mL of the glycoprotein asialofetuin (ASF) in PBS, overnight at room temperature. After washing with PBST, blocking was performed with 200 µL/well of blocking solution (1% [*w*/*v*] BSA in PBST) for 1 h at 37 °C. Purified ML-II protein was plated starting at a top concentration of 10 µg/mL and diluted 3 fold (8 dilutions in all), followed by incubation for an additional 2 h at 37 °C. The ML-II protein bound to ASF was detected with 5F5 anti-mistletoe A chain antibodies (1:1000; SIFIN, Germany) and secondary anti-mouse IgG-HRP (1:5000; Southern biologics, Tallahassee, FL, USA). The wells were washed with an ELISA automated plate washer (Thermo Scientific, Waltham, MA, USA), and the samples were incubated with KPL SureBlue™ substrate (SeraCare, Milford, MA, USA) for 20 min, in the dark at room temperature. The reaction was stopped by the addition of 50 µL/well of 1 M H_2_SO_4_. The plates were read at 450 nm on a Biotek ELISA plate reader. 

### 4.12. Cell Lines

Human large cell carcinoma NCI-H460 and human adenocarcinoma A549 cell lines were obtained from the ATCC (Manassas, VA, USA) and maintained in Dulbecco’s modified Eagle’s medium (DMEM) supplemented with penicillin (50 U/mL), streptomycin (50 mg/mL), and 10% fetal bovine serum (FBS; Gibco, USA) at 37 °C in a humid environment containing 5% CO_2_. 

### 4.13. MTS Cell Viability Assay 

The MTS assay was performed to determine the viability of the A549 and H460 cells treated with ML-II. The cells were incubated in 96-well tissue culture plates (Corning, NY, USA) at a density of 10 × 10^3^ cells/well in 200 µl at 37 °C in a humidified, 5% CO_2_ atmosphere incubator. After 24 h of incubation, the cells were treated with ML-II or vehicle (PBS) starting at a concentration of 50 µg/mL. The cytotoxic effects were measured after 96 h of incubation. The media were removed and replaced with PBS supplemented with 4.5 g/L glucose, which was followed by the addition of 20 µL of the MTS solution to every well and incubation for 1–4 h at 37 °C [43]. The production of formazan by live cells was determined by measuring the OD at 490 nm. Cell viability was calculated as follows: % viability = absorbance of treated/absorbance of untreated × 100. In addition, morphological changes were assessed under an EVOS inverted microscope (Thermo Fisher, Waltham, MA, USA).

### 4.14. Cell Apoptosis Assay 

Cells (2 × 10^5^) were seeded into 6-well plates and treated with 10 μg/mL of ML-II or vehicle (PBS) for 24, 48, and 72 h, respectively. After treatment, the cells were submitted to annexin V-488A and propidium iodide (PI) (Biotium, Fremont, CA, USA) double staining, according to the modified annexin V-448A and PI apoptotic assay protocols [44]. Briefly, after 24 h of culture, cells were treated with ML-II (10 µg/mL), 0.1 M d-galactose, ML-II (10 µg/mL) + 0.1 M d-galactose (inhibition study), or the vehicle control (PBS). Then, at different time points, cells were trypsinized and pelleted at 335× *g* for 10 min. Then, the cells were resuspended in 1× annexin V binding buffer and stained, first, with annexin V-448A in the dark for 15 min at room temperature, and then PI was added. Next, the cells were washed and fixed with 1% formaldehyde solution for 10 min on ice. After two washes with PBS, the cells underwent treatment with RNase A for 15 min at 37 °C. Approximately 3000–5000 cells in each sample were analyzed on a BD FACS Calibur flow cytometer, and apoptotic cells were detected. The percentages of cells positive for annexin V-448A and PI were reported inside the quadrants. The data were processed and analyzed with FlowJo v10 (FlowJo LLC, Ashland, OR, USA). Treatment groups were compared by one-way or two-way ANOVA, with a P value of 0.05 as the threshold of significance.
